# Primary care prediction of hip and knee replacement 1–5 years in advance using Temporal Graph-based Convolutional Neural Networks (TG-CNNs)

**DOI:** 10.1093/rheumatology/keaf185

**Published:** 2025-04-03

**Authors:** Zoe Hancox, Sarah R Kingsbury, Philip G Conaghan, Andrew Clegg, Samuel D Relton

**Affiliations:** School of Computing, University of Leeds, Leeds, UK; Leeds Institute of Rheumatic and Musculoskeletal Medicine, University of Leeds, Leeds, UK; Leeds Biomedical Research Centre, NIHR, Leeds, UK; Leeds Institute of Rheumatic and Musculoskeletal Medicine, University of Leeds, Leeds, UK; Leeds Biomedical Research Centre, NIHR, Leeds, UK; Academic Unit for Ageing and Stroke Research, Bradford Institute for Health Research, Bradford, UK; School of Computing, University of Leeds, Leeds, UK

**Keywords:** hip, knee, joint replacement, risk prediction, electronic health records, graphs

## Abstract

**Objective:**

This study aimed to predict the risk of requiring a primary hip or knee replacement 1 and 5 years in advance, using clinical codes.

**Methods:**

Primary care clinical codes, sourced from ResearchOne Electronic Health Records between 1999 and 2014, were used to represent patient pathways prior to hip or knee replacement. Patient records were used to train and test models for hip or knee replacement, 1 and 5 years in advance. Temporal graphs were constructed from clinical codes to predict hip and knee replacement risk, where nodes are clinical codes, and edges are the time between primary care visits. Hip and knee replacement cases were matched to controls by age, sex and Index of Multiple Deprivation. The model was validated on unseen data, with performance measured using area under the receiver operator curve (AUROC), calibration and area under the precision recall curve (AUPRC), recalibrating for class imbalance.

**Results:**

For knee replacement prediction, AUROC was 0.915 (95% CI 0.914, 0.916) (1 year) and 0.955 (95% CI 0.954, 0.956) (5 years) with AUPRCs of 0.353 (95% CI 0.302, 0.403) and 0.442 (95% CI 0.382, 0.503), respectively. For hip replacement prediction, AUROC was 0.919 (95% CI 0.918, 0.920) (1 year) and 0.967 (95% CI 0.966, 0.968) (5 years), with AUPRCs of 0.409 (95% CI 0.366, 0.451) and 0.879 (95% CI 0.833, 0.924), respectively.

**Conclusion:**

Hip and knee replacement risk can be predicted up to 5 years in advance, with a temporal-graph based artificial intelligence model achieving the best performance. This may be used for planning preventative treatment or triaging patients.

## Graphical abstract

**Figure:**
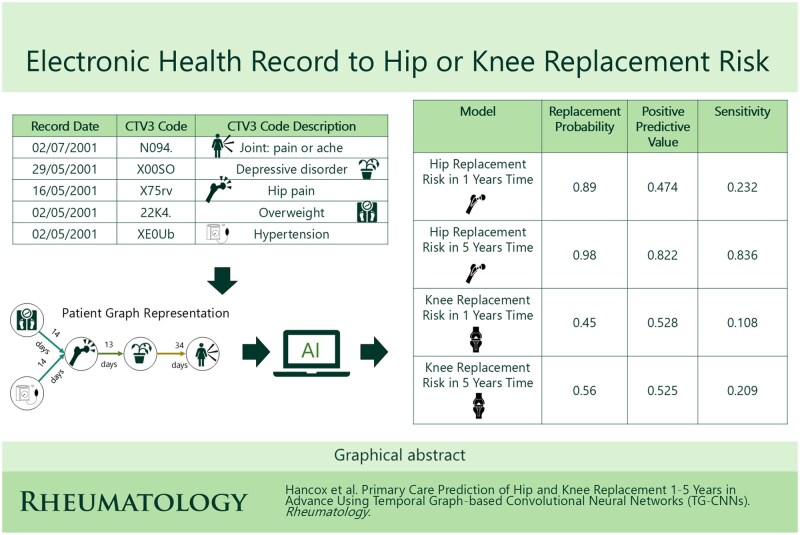

Rheumatology key messages

- Primary care visit records can be used to predict an individual’s future joint replacement risk.
- Hip and knee replacement risk can be predicted 5 years in advance using patient data.
- The temporal graph-based model provided the best calibration for hip and knee replacement risk prediction.

## Introduction

OA is associated with decreased physical activity, pain and increased morbidity [1]. Some 32.5% of those with hip OA and 16.7% with knee OA eventually require joint replacement [2]. OA presents a major challenge for healthcare, with musculoskeletal issues accounting for 20% of primary care consultations [3, 4]. In 2015, £10 billion was spent on musculoskeletal treatment and care [5].

In 2014, the UK National Joint Registry recorded 83 125 hip replacements, with £438.9 million being spent by the UK National Health Service (NHS) on primary hip replacements [6]. In 2009, OA was the fourth most common reason for hospitalization [7]; 25% of adults are expected to develop symptomatic OA [7]. Pressure on hospitals will rise and with more people in pain as OA prevalence increases with ageing populations [6, 8]. From 2003 to 2014, 708 311 primary total hip replacements and 772 818 primary knee replacements were performed in the UK, with OA accounting for 93% and 96% of these procedures, respectively [6].

Clinical prediction models can estimate an individual’s risk of future medical events, prior to any physical tests. Predicting the need for future joint replacement is valuable for resource planning, and clinician decision-making. For example, exercise therapy can reduce hip replacements by 44% [9], and early interventions can improve patient quality of life and lower surgery rates.

Previously, the Temporal Graph-based Convolutional Neural Network (TG-CNN) methodology was introduced for the prediction of hip replacement risk 1 year in advance using Clinical Terms Version 3 (CTV3) codes and demographics from ResearchOne data [10], which includes code used in this study. In this paper, four separate models are created for hip and knee prediction, predicting replacement risk 1 and 5 years in advance.

The Index of Multiple Deprivation (IMD) score was included as an indicator of sociodemographic status, based on research showing that individuals with less deprivation were less likely to have hip replacements [2]. Additionally, age is a critical predictor, with knee replacement risk peaking in 60- to 70 year olds, and hip replacements peaking at 55 years [6, 11]. Sex is also significant—women account for 60% of hip replacements and 57% of knee replacements [6].

The aim of this paper is to predict hip or knee replacement risk 1 and 5 years in advance, using CTV3 codes constructed as temporal graphs for prediction using the TG-CNN model. The results from this model are compared against current state-of-the-art models.

## Methods

An electronic health record (EHR) was defined as an individual patient’s record, consisting of primary care CTV3 codes with timestamps. Each visit (time step *k*) was defined as a unique date. Sequences of CTV3 codes were transformed into individual patient graph representations, which were used to predict hip and knee replacement risk.

CTV3 codes were used as graph nodes, while edges captured time intervals between code occurrences, measured in months. Each of the 512 most frequently used CTV3 codes and six prescription types [acute and repeat prescriptions of: opioids, non-opioid analgesics (NOAs) and NSAIDs] were mapped to a unique node.

These graphs were then inputted into the TG-CNN model to process CTV3 codes and time intervals from EHRs. For full technical details of the TG-CNN model and methodology, refer to previous work [10] or for the model architecture see Supplementary Fig. S1, available at *Rheumatology* online.

A systematic search was carried out which answered the following question: ‘What predictive methods are used to assess an individual’s future risk of primary hip or knee replacement before secondary care referral?’ Further methodology can be found in the Supplementary Material, available at *Rheumatology* online.

### Dataset

NHS primary care data from ResearchOne were used, comprising clinical and administrative data from 151 565 patients who attended healthcare practices in England using the SystmOne primary care EHR system. Patients had their first record of joint pain clinically coded between 1 April 1999 and 31 March 2014 [12]. Included patients had a combined total of 243 700 primary care visits, with patients having an average of 1.36 visits and a maximum of 10 CTV3 codes recorded per visit.

### Patient inclusion criteria

Patients were aged 40–75 years at the start of the analysis period (average age 56.67 ± 9.37 years). This range was chosen as these patients are likely to present with musculoskeletal symptoms. Younger patients usually have less joint wear and only need replacements due to rare conditions or accidents. Focusing on those over 40 years of age enhances the accuracy and clinical utility of prediction models by targeting the population most at risk for joint replacements and who are most likely to benefit from early identification and intervention. This approach reduces variability and improves generalizability.

Patients were required to have had at least two primary care visits within either 1 or 5 years prior to undergoing a hip or knee replacement (partial or full). Without two visits an EHR TG-CNN graph-representation cannot be established. Clinician-selected CTV3 codes for primary hip and knee replacements were used to identify the outcome, with the incident date defined as the first occurrence of one of these codes (Supplementary Tables S1 and S2, available at *Rheumatology* online). The dataset exhibited an imbalanced case–control split, reflecting a common challenge in clinical research where, despite the high prevalence of the underlying condition, the proportion of patients undergoing hip or knee replacement is relatively small.

Patients were removed if they had a CTV3 code referring to a revision/modification of a hip or knee replacement before a code suggesting primary replacement. If a patient had a revision of a joint but no primary joint replacement they were removed from the control group. CTV3 hospital referral codes associated to hip or knee replacement were excluded to prevent target leakage. See Supplementary Fig. S2, available at *Rheumatology* online for the inclusion flowchart for patients included the hip 1 year in advance dataset.

### Training and test set formation

A Dell Precision 3650 machine, with an 11th generation intel 8 core i7-11700, DDR4 main memory with 64GB of RAM, a NVIDIA GeForce RTX 3090 24GB GPU was used to train these models.

Test datasets were created for each of the four models, with a random 10% selected for the test data. The remaining data was assigned to the training set, where each joint replacement patient was matched to a control. Exact 1:1 matching was performed for age, sex and IMD to mitigate their confounding influence on joint replacement risk prediction. Matching was limited to three variables to ensure sample size was not significantly affected by the combinatorics, which would reduce model performance. While exact matching ensures balanced distributions of these variables between cases and controls, it can also limit model generalizability by restricting variability within the training data. To address this, model recalibration was performed after training and optimization—each model was recalibrated using a logistic regression generalized linear model to adjust risk probabilities for the shift from balanced to unbalanced case–control data. The model was trained on the balanced dataset, using 5-fold cross-validation, with one-fifth of the patients iteratively used as holdout/validation data to evaluate model performance by splitting the patients randomly into five groups. Training on a balanced dataset, where each class was equally represented, ensured that the model learned to give equal importance to all classes, preventing majority class bias. Complete case analysis was employed to estimate the marginal effect of hip or knee replacement [13], under the assumption of no mortality to reduce competing risks as the mortality rate was 0.007%. If a patient dies before undergoing joint replacement, including them could introduce noise or bias as the true replacement outcome is unknown. Similarly, risk estimates could be skewed if mortality is included, particularly if death is associated with factors linked to joint replacement risk, such as servere comorbidities.

### Model predictors

To forecast replacements, patient records from 1999 until 1 or 5 years before the primary replacement date were collected, depending on the prediction timeframe.

Code usage in the ResearchOne dataset was analysed, counting occurrences across patients and visits. The top 512 CTV3 codes, representing 99.46% of recorded events and 21.76% of unique codes (512/2353), were used for graph construction. Medications were appended as six predictors using British National Formulary codes, grouping drugs into opioids (04.07.02), NOAs (04.07.01) and NSAIDs (10.01.01), subgrouped into acute and repeat prescriptions. Age, sex and IMD demographic predictors were used. With the inclusion of six prescription types and three demographics, the total number of predictors was 521. No standard method exists for calculating the sample size, so it was maximized from the available dataset, and overfitting checks were performed.

In previous work, an ablation study was carried out to test various component effects on the model’s performance [10]. The best model from this study (TG-CNN w/o *ℓ*_2_) was used and prescription records included. Including repeat and non-repeat drug prescriptions of NSAIDs, opioids and NOAs was useful, because although they could be prescribed for numerous reasons, they are a good indication of pain levels in patients.

Events occurring further in the past may have a diminishing impact on recent events. Previously analysis was limited to the 100 most recent primary care visits per patient to manage computational load. In this paper, the number of visits (*k*_max_) used in this model was explored, it was hypothesized that increasing the number of visits would enable the model to learn more as the prescription data would not outnumber the other nodes in the graphs. To determine the optimum number of visits, the distribution of time covered by the full patient history within the analysis period was investigated, observing the difference between the number of years covered in the full history *vs* the last 100, 150 and 200 primary care visits.

### Model evaluation

The hip and knee prediction models were tested, both 1 and 5 years prediction in advance, with and without prescriptions. Random forests, logistic regression and recurrent neural network (RNN) models were used as comparison models. Time was not included in comparison models due to their limitations.

Calibration can be used to assess risk estimate reliability, by comparing the agreement of the true number of hip/knee replacements to the predicted number. It is calculated by fitting a logistic regression model of the observed replacement outcomes against the predicted probabilities. A slope (C-slope) of 1 indicates perfect calibration, while values <1 or >1 suggest overfitting or underfitting, respectively.

Discriminative performance was assessed using area under the receiver operating characteristic curve (AUROC), which evaluates how well the model assigns higher risks to case patients and lower risks to controls. A perfect model has an AUROC of 1, while a poorly performing model scores near 0.5. Area under the precision recall curve (AUPRC) is better suited for imbalanced datasets. With low positive class prevalence, AUPRC is a more suitable metric than AUROC, as it focuses on identifying positive cases rather than balancing both classes. Meaningful performance is indicated when AUPRC exceeds the positive class prevalence; for instance, an AUPRC above 0.05 suggests reasonable prediction if 5% of the population undergoes hip replacement surgery.

Confidence intervals for C-slope, AUROC and AUPRC were calculated using Wald’s confidence interval calculation.

Data not used in training was used to compare recalibrated model results. Due to the lack of primary care location information, cluster analysis was not performed. AUPRC, AUROC and calibration curves for sex and IMD quintile subgroups and descriptive analysis were provided for the TG-CNN models.

The TRIPOD-AI statement [14] was followed for reporting of model methodology and evaluation (see the Supplementary Material, available at *Rheumatology* online for the completed checklist).

## Ethics

Approval for the study was obtained from the School of Medicine Research Ethics Committee (SoMREC) at the University of Leeds (reference: SoMREC/13/079) and the Research Project Committee at ResearchOne (project number: 201428378A).

## Results

### Study population

The hip models used 33 074 patient records (10 206 hip replacements, 30.9%), while the knee models used 30 492 records (8723 knee replacements, 28.6%).

Patients living in more deprived regions (IMD 1) visited primary care on average less frequently (54.6 ± 56.5) than people living in less deprived regions (IMD 5) (82.9 ± 73.9).

Table 1 displays further characteristics of the included cohort data. Supplementary Table S3, available at *Rheumatology* online provides details of characteristics grouped by training and testing dataset, with and without prescriptions.

**Table 1.** Replacement prediction model cohort characteristics

	Hip 1 year	Hip 5 years	Knee 1 year	Knee 5 years
Characteristic	(*N* = 33 074)	(*N* = 24 732)	(*N* = 30 492)	(*N* = 24 370)
Sex: female [*N* (%)]	20 562 (62.2)	15 252 (61.7)	18 482 (60.6)	14 837 (60.9)
Hip/knee replacements [*N* (%)]	10 206 (30.9)	5857 (23.7)	8723 (28.6)	5543 (22.7)
Age, years				
Mean (s.d.)	72.5 (9.9)	71.4 (10.2)	72 (9.8)	71 (9.9)
Median (min, max)	73 (53, 89)	71 (53, 89)	72 (53, 89)	71 (53, 89)
Mode	88	88	88	66
Age at replace, years				
Mean (s.d.)	69.2 (8.5)	70.6 (8.5)	68.9 (8.1)	69.6 (8.2)
Median (min, max)	70 (43, 90)	71 (48, 90)	69 (46, 89)	70 (47, 89)
Mode	77	77	75	75
BMI [*N* (%)]				
Severely obese	1017 (3.1)	792 (3.2)	1205 (4.0)	943 (3.9)
Obese	9480 (28.7)	7062 (28.6)	9538 (31.3)	7474 (30.7)
Overweight	12 208 (36.9)	9101 (36.8)	10 948 (35.9)	8775 (36.0)
Healthy weight	8193 (24.8)	6052 (24.5)	6855 (22.5)	5578 (22.9)
Underweight	544 (1.6)	416 (1.7)	437 (1.4)	367 (1.5)
Missing	1632 (4.9)	1309 (5.3)	1509 (4.9)	1233 (5.1)
IMD, quintile [replacements/total (%)]				
1 (most deprived)	2749/8301 (33.1)	1588/6104 (26.0)	2021/7007 (28.8)	1280/5611 (22.8)
2	2546/7836 (32.5)	1452/5753 (25.2)	2035/6966 (29.2)	1282/5515 (23.2)
3	2246/7125 (31.5)	1267/5128 (24.7)	1937/6544 (29.6)	1234/5172 (23.9)
4	1493/5116 (29.2)	852/3876 (22.0)	1435/5002 (28.7)	919/4057 (22.7)
5 (least deprived)	1172/4696 (25.0)	698/3871 (18.0)	1295/4973 (26.0)	828/4015 (20.6)
Events recorded				
Mean (s.d.)	58.0 (91.8)	61.9 (97.5)	66.5 (99.8)	63.9 (95.4)
Median (min, max)	23.0 (2, 1890)	24.0 (2, 1255)	29.0 (2, 1494)	29.0 (2, 1890)
Mode	2	2	2	2

The BMI statistics are derived from the last recorded BMI measurement of each patient. The first recorded Index of Multiple Deprivation (IMD) value for each patient was used, given its stability over time.

### EHR coverage

**Figure 1.**
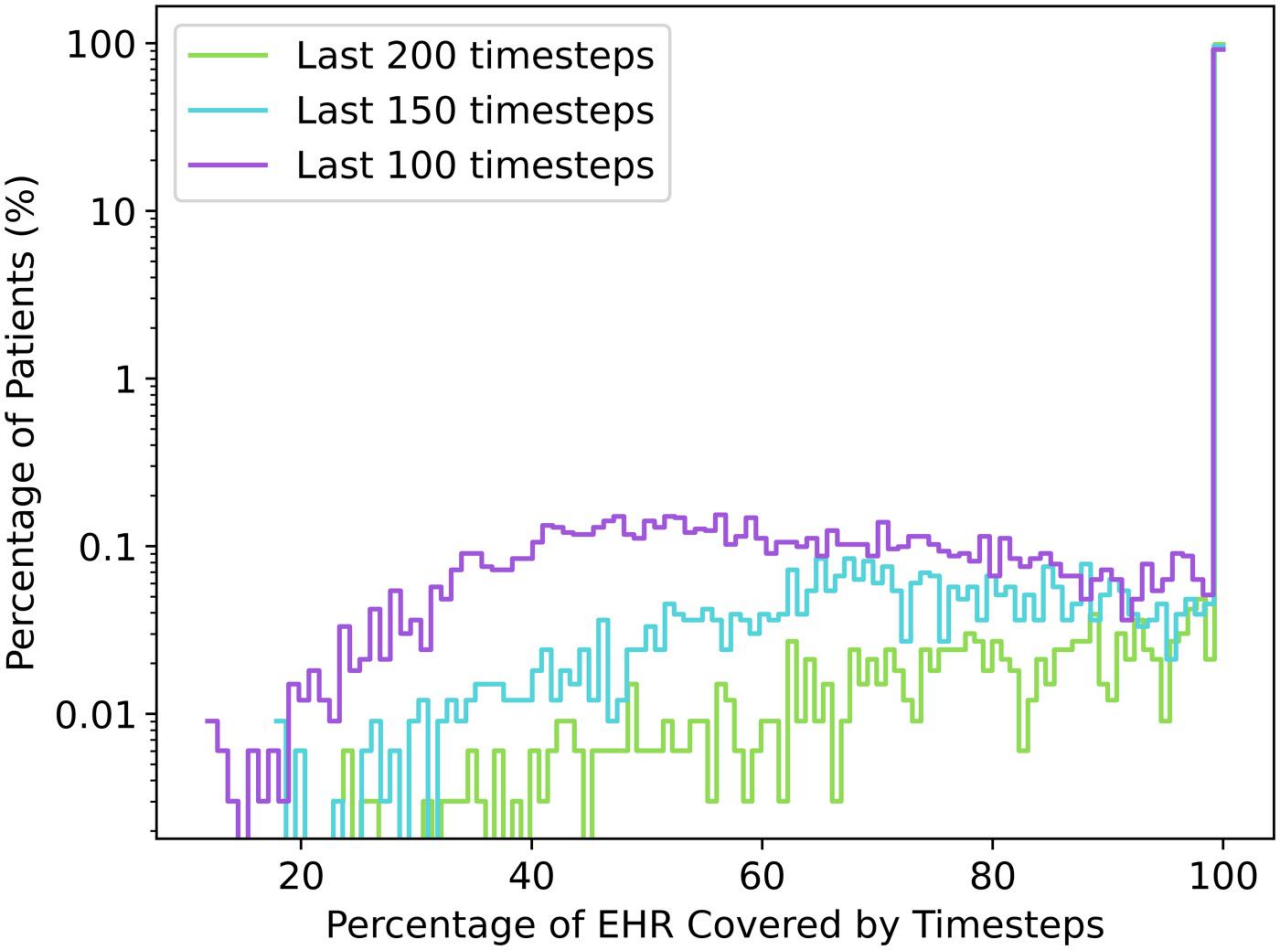
Percentage of EHR covered by including different numbers of visits (100 150 and 200) 1 year in advance for hip replacement prediction. EHR: electronic health record.

**Figure 2.**
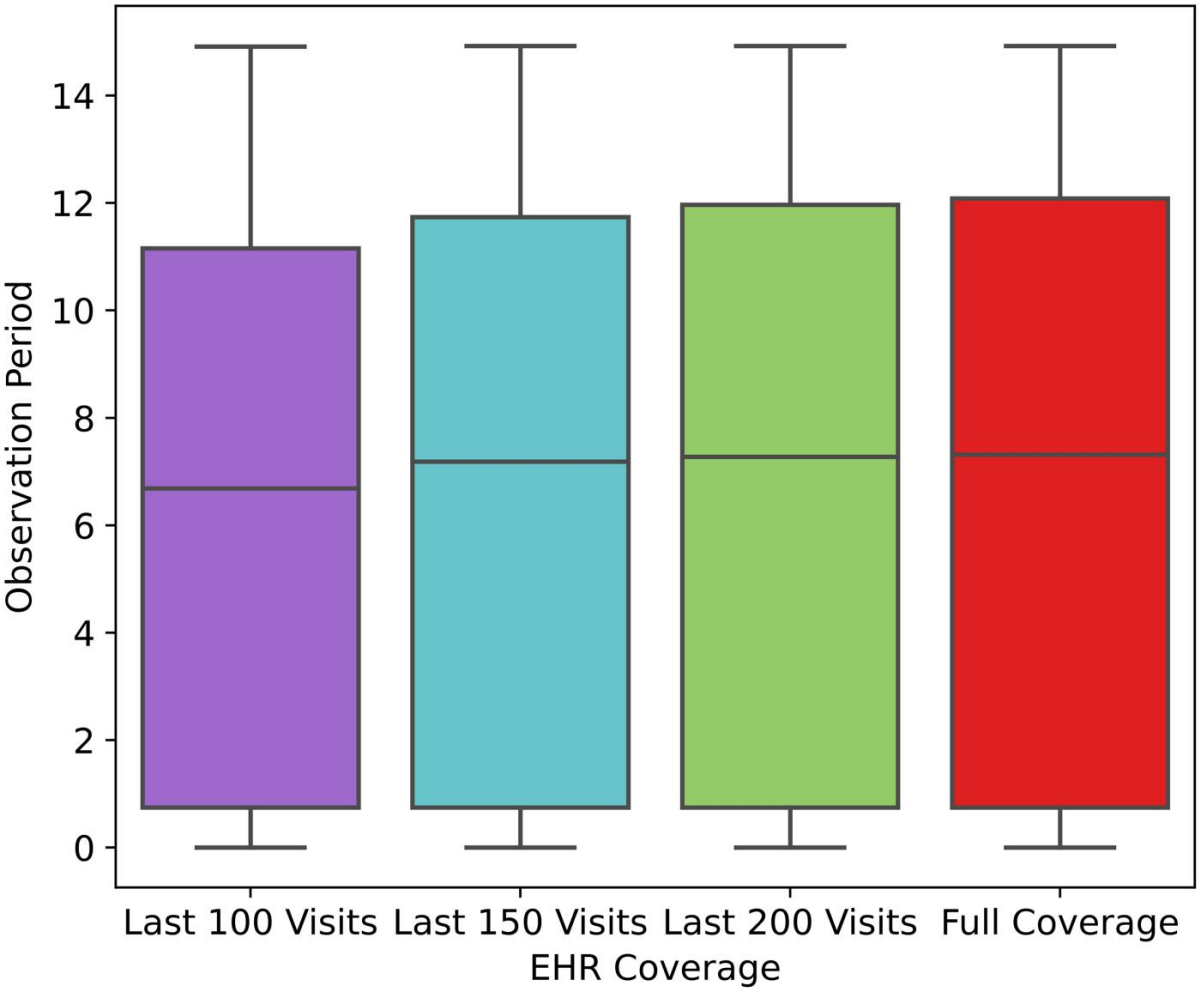
Observation period for 1 year in advance hip replacement prediction, exploring coverage based on different numbers of visits. The distribution of the length of time observed within 15-year EHR data is shown when using the last 100, 150 and 200 primary care visits, compared with full coverage. EHR: electronic health record.

Fig. 1 shows that *k*_max_ = 200 provided optimal coverage, with all patients having at least 25% of their records covered and <1% with incomplete EHR coverage (1999–2014). Fig. 2 indicates patients had, on average, over 6 years of EHRs during this period.

Using the last 200 visits, coverage was 98.71% for hip 1-year data, 98.38% for hip 5-year data, 98.55% for knee 1-year data and 98.50% for knee 5-year data.

### Model performances

**Figure 3.**
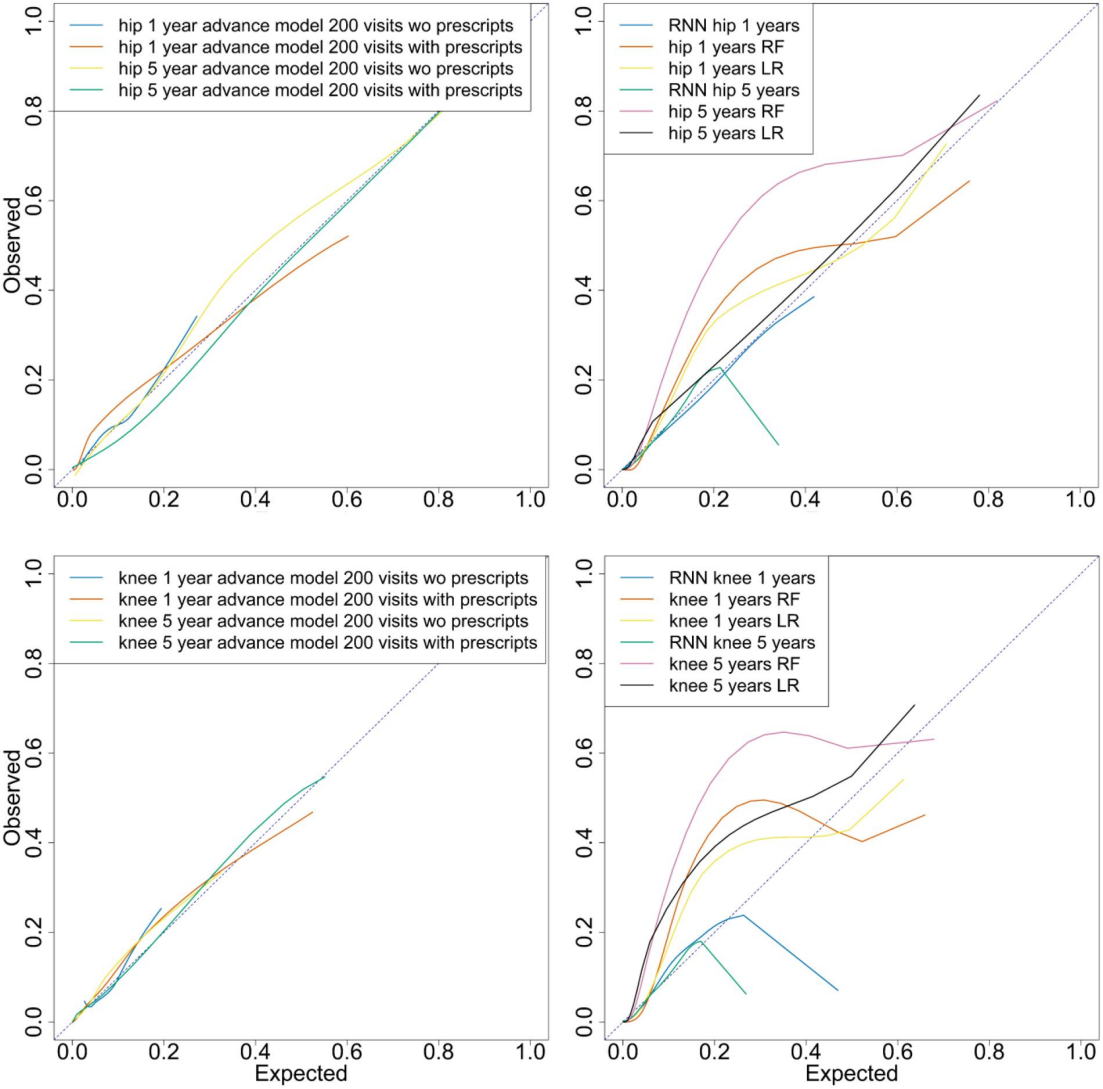
Calibration curves for all TG-CNN (left) and baseline models (right) for both hip (top 2) and knee (bottom 2) replacement models, 1 and 5 years in advance. TG-CNN: Temporal Graph-based Convolutional Neural Networks; RF: random forest; RNN: recurrent neural network; LR: logistic Regression.

Table 2 shows the performance metrics for each of the TG-CNN and comparison models. Fig. 3 shows the calibration curves for all the models in Table 2. Calibration curves are important to plot to show accuracy of predicting model correctness [15]. The calibration curves showed that the TG-CNN models fitted the population well, whilst the comparison models had poor calibration. All TG-CNN models without prescriptions, except the hip 5-year advance model, predicted no patients would need a replacement (positive predictive value = 0). The hip 5-year advance model had the highest sensitivity among all models. Logistic regression outperformed all models except the hip 5-year advance model. The logistic regression and random forest models underestimated the risk of needing hip or knee replacement, whilst the RNN model overestimated the risk. The hip 5-year in advance prediction logistic regression model had good calibration, however the other comparison models with C-slope values close to 1 were largely due to non-linear calibration. Supplementary Figs S3 and S4, available at *Rheumatology* online show sensitivity and specificity scores for each probability threshold for the TG-CNN and logistic regression models.

**Table 2.** AUPRC, C-slope and AUROC results for the models on the unseen test data set with bold values depicting the row with the best results for each replacement and year model group

Model	AUPRC (95% CI)	C-slope (95% CI)	AUROC (95% CI)	PPV	Sensitivity	Specificity
Hip 1 year in advance						
TG-CNN with prescriptions	0.409 (0.366, 0.451)	0.976 (0.970, 0.983)	0.919 (0.918, 0.920)	0.474	0.232	0.981
TG-CNN w/o prescriptions	0.192 (0.164, 0.219)	1.075 (1.065, 1.084)	0.734 (0.732, 0.736)	0.000	0.000	1.000
RNN with prescriptions	0.293 (0.260, 0.325)	1.050 (1.040, 1.061)	0.895 (0.894, 0.896)	0.100	0.002	0.999
RF with prescriptions	0.431 (0.389, 0.472)	**0.996 (0.990, 1.001)**	0.933 (0.932, 0.933)	0.497	0.194	0.986
LR with prescriptions	**0.477 (0.428, 0.525)**	0.995 (0.989, 1.001)	**0.938 (0.937, 0.938)**	**0.586**	**0.255**	0.987
Hip 5 years in advance						
TG-CNN with prescriptions	**0.879 (0.833, 0.924)**	1.047 (1.034, 1.061)	0.967 (0.966, 0.968)	**0.822**	**0.836**	0.947
TG-CNN w/o prescriptions	0.762 (0.704, 0.820)	1.059 (1.046, 1.072)	0.913 (0.911, 0.915)	0.752	0.655	0.937
RNN with prescriptions	0.184 (0.158, 0.209)	1.073 (1.059, 1.088)	0.894 (0.893, 0.895)	0.000	0.000	0.999
RF with prescriptions	0.567 (0.507, 0.627)	0.969 (0.961, 0.978)	0.969 (0.968, 0.969)	0.726	0.319	0.995
LR with prescriptions	0.620 (0.564, 0.676)	**1.021 (1.010, 1.032)**	**0.973 (0.973, 0.973)**	0.725	0.336	0.994
Knee 1 year in advance						
TG-CNN with prescriptions	0.353 (0.302, 0.403)	**0.997 (0.989, 1.005)**	0.915 (0.914, 0.916)	0.528	0.108	0.994
TG-CNN w/o prescriptions	0.140 (0.115, 0.165)	1.094 (1.077, 1.110)	0.661 (0.658, 0.664)	0.000	0.000	1.000
RNN with prescriptions	0.193 (0.171, 0.216)	0.901 (0.892, 0.910)	0.864 (0.863, 0.866)	0.000	0.000	0.996
RF with prescriptions	0.335 (0.294, 0.376)	0.953 (0.948, 0.958)	0.925 (0.924, 0.926)	0.392	**0.128**	0.987
LR with prescriptions	**0.374 (0.326, 0.423)**	0.965 (0.959, 0.972)	**0.928 (0.927, 0.929)**	**0.550**	0.126	0.994
Knee 5 years in advance						
TG-CNN with prescriptions	0.442 (0.382, 0.503)	0.980 (0.970, 0.991)	0.955 (0.954, 0.956)	0.525	0.209	0.992
TG-CNN w/o prescriptions	0.238 (0.199, 0.277)	0.990 (0.981, 0.999)	0.856 (0.853, 0.858)	0.000	0.000	1.000
RNN with prescriptions	0.146 (0.126, 0.166)	0.951 (0.938, 0.965)	0.860 (0.859, 0.862)	0.000	0.000	1.000
RF with prescriptions	0.467 (0.410, 0.524)	**0.997 (0.989, 1.004)**	0.963 (0.963, 0.964)	0.649	0.205	0.995
LR with prescriptions	**0.500 (0.438, 0.561)**	0.975 (0.966, 0.984)	**0.965 (0.965, 0.966)**	**0.680**	**0.236**	0.995

Random forests and logistic regression models contained demographics, prescriptions and CTV3 codes as predictors. RNN models contained prescriptions and CTV3 codes. AUPRC thresholds based on prevalence for each dataset are as follows: hip 1 year = 0.069, hip 5 years = 0.227, knee 1 year = 0.059, knee 5 years = 0.041 (AUPRC scores lower than their respective threshold can be deemed as uninformative models), where for example, the AUPRC threshold for the hip 1 year model was selected at 0.069 as 6.9% of the patient’s data used in this model had a hip replacement procedure. AUPRC: area under the precision recall curve; AUROC: area under the receiver operator curve; PPV: positive predictive value; TG-CNN: Temporal Graph-based Convolutional Neural Networks; RF: random forest; LR: logistic regression; CTV3: Clinical Terms Version 3.

All TG-CNN models had non-overlapping CIs when comparing AUPRC with and without prescriptions, suggesting a significant difference when including prescriptions in the model. However, C-slope confidence intervals slightly overlapped in 5-year advance models for hip and knee replacement risk. Two-sample t-tests yielded *P*-values of 0.0 (95% confidence) when comparing AUPRCs and AUROCs with and without prescriptions, and between logistic regression and TG-CNN models with prescriptions, confirming significant differences. See Supplementary Table S4, available at *Rheumatology* online for details.

**Figure 4.**
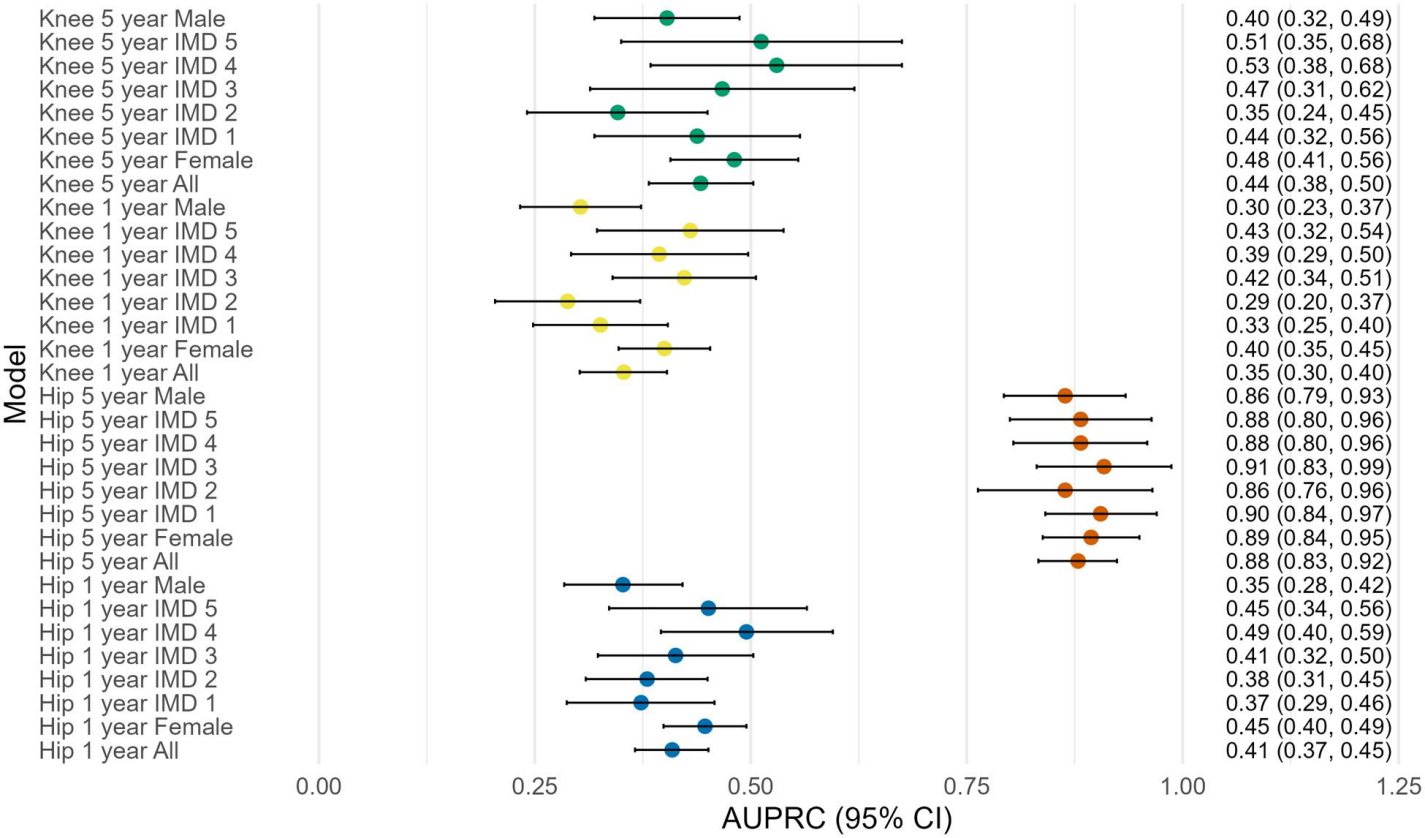
Forest plot showing AUPRC scores and 95% CIs for each of the TG-CNN models (with prescription data included) and subgroups. AUPRC: area under the precision recall curve ;TG-CNN: Temporal Graph-based Convolutional Neural Networks; IMD: Index of Multiple Deprivation.

Subgroup analysis showed females had higher AUPRC scores (0.030–0.097) than males across all TG-CNN models. IMD 4 groups performed best, while IMD 1 and 2 scored lower. The hip 5-year model had the best generalizability, with all subgroups scoring above 0.864. AUPRC ranges were smallest in the knee 1-year model (0.288–0.430). See Fig. 4 and Supplementary Figs S5 and S6, available at *Rheumatology* online for details.

Five papers were identified as relevant to the systematic search research question [16–20]. The model with the best performance for hip and knee replacement risk was the Oxford Knee and Hip Score (OKHS) model [16], with AUROCs of 0.87 and 0.83 for hip and knee prediction. The TG-CNN model outperformed the current state of the art risk prediction tools for hip and knee replacement by AUROCs of 0.1 and 0.13, respectively. Further details of the search results can be found in the discussion and Supplementary Materials, available at *Rheumatology* online.

## Discussion

This work shows that hip and knee replacement risk can be predicted 1–5 years in advance using routine primary care data from up to 200 patient visits. Predicting replacement risk 5 years in advance outperformed 1-year predictions in all models except the RNN knee model. The 5-year hip model may have performed better due to a higher hip replacement prevalence (0.227) compared with the 1-year group (0.069). The TG-CNN produced the best calibration curves for risk prediction.

The 5-year prediction models outperformed expectations despite fewer data and fewer patterns, possibly due to reduced noise, stronger long-term predictors, less overfitting or a clearer signal from long-term health deterioration.

Subgroup analysis showed the models performed slightly worse on males, possibly due to the higher prevalence of joint replacements in females. Subgroup analysis revealed that patients in the IMD 4 group generally had better model performance, while those in more deprived regions showed worse performance, despite higher replacement prevalence in these groups. The data showed more frequent visits and larger numbers of records in less deprived populations, possibly due to limited access to primary care in more deprived areas, resulting in fewer EHR data [21, 22].

While subgroup analyses were conducted across age, sex and IMD, other biases may be present in the training data that could affect the fairness of the model across populations. These biases can arise from data collection methods, representation of different groups and model characteristics. The distribution of age in the training data may not reflect the true population. If age groups are over or underrepresented the model may perform differently across age subgroups, leading to biased predictions. If sex imbalance exists, the model may be biased and favour the overrepresented sex. If certain deprivation levels are underrepresented, or the diversity of socioeconomic status is not captured sufficiently, the model may struggle to predict fairly across IMD groups. Further work is required to monitor and address model prediction biases, particularly in new out-of-sample data.

Resampling to correct for class imbalance can over-estimate risk and may not improve re-calibration in some models. However, the TG-CNN model showed improved performance after re-calibration on out-of-sample imbalanced data, despite being trained on balanced data.

Logistic regression was used to recalibrate the model, as this model is simple and effective to transform predicted values into calibrated probabilities, even when applied to more complex models such as TG-CNN. However, recalibration with logistic regression assumes that the predicted probabilities are linearly related to the logits that may not always capture complex data relations. Additionally, while re-calibration can enhance calibration, it does not affect discrimination [23].

### Related work

The OKHS screens patients for surgical or non-surgical hip and knee referrals using brief surveys on pain and mobility, achieving AUROCs of 0.83 for knee referrals and 0.87 for hip referrals [16].

A Fine and Gray competing risk model estimates the 5-year risk of total knee replacement using factors like age, previous knee replacement, non-replacement knee surgery, OA prescriptions, comorbidity index and mental health, achieving an AUROC of 0.67 [17].

Cumulative incidence equations estimated the 10-year risk of hip or knee replacement in individuals with OA and/or pain, yielding an AUROC of 0.72 for hip replacement and 0.78 for knee replacement. Key predictors for knee replacement included oral NSAIDs, opioid prescriptions, intra-articular injections and prior arthroscopic knee surgery, while a history of hip injury was a strong indicator for future hip replacement risk [18].

A knee replacement risk model for OA patients using random survival forests achieved an AUROC of 0.807, incorporating predictors like sex, age, education, BMI, occupational activity, smoking, medical history, pain medication, knee injury and surgery, knee pain and stiffness, and use of a walking aid. Interpretability methods identified pain medication, age, surgery, diabetes and BMI as the top risk factors for knee replacement [19].

Total knee replacement risk was modelled using Cox proportional hazards with primary and secondary care predictors in patients with recent knee pain. The secondary care model, based on demographics, knee pain, analgesics, WOMAC pain score and Kellgren–Lawrence grade, outperformed the primary care model, which included additional features like BMI and knee arthroscopy history, achieving an AUROC of 0.87 *vs* 0.79 [20].

The TG-CNN models improved hip and knee replacement risk prediction by incorporating a broad range of predictors, including IMD score, CTV3 codes and mental health factors, extending beyond OA patients or those with recent joint pain. Unlike previous models, it covers both partial and total replacements and captured temporality using graphs, allowing repeated predictors like painkiller prescriptions to estimate pain severity. TG-CNN models also offer patient-specific trajectory importance, enhancing explainability and supporting clinical decision-making. This comprehensive approach reduces the likelihood of missing patients with atypical pathways to replacement.

### Limitations

This model has not undergone external validation as it has not been tested on external datasets for generalizability [24], though re-calibrated out-of-sample data suggest good performance on new cases. The dataset lacked ethnicity, a valuable predictor for hip replacement, which could improve explainability for clinicians and patients. Only CTV3 codes and limited demographics were used, while including imaging data might enhance predictions [20, 25]. However, using CTV3 codes minimizes reliance on costly methods, enabling earlier predictions for intervention at the primary care level. Prior to specialty joint clinic referral, a patient will unlikely have joint images in their EHR, limiting that data type inclusion, however other clinical data such as pain scores could be beneficial to improving joint replacement risk accuracy. The dataset’s regional coverage and size were limited compared with datasets such as Clinical Practice Research Datalink. These models were also trained on recorded replacement dates, rather than optimum surgery times, which meant that waiting times were not considered. CTV3 codes are no longer used, however this model could be retrained or revalidated in future research with alternative clinical codes using the same methodology as provided in this paper.

While the 512 CTV3 codes covered 99.46% of recorded clinical events, we did not assess the impact of using fewer codes. However, given the high coverage, rerunning the analysis was unlikely to provide additional insights. Conducting a meta-analysis of various interventions could help assess their costs and associated risks, potentially identifying more cost-effective approaches. Involving patients and public for risk grouping, alongside performing decision curve analysis, could further demonstrate whether these strategies are viable for reducing healthcare costs. The TG-CNN model could assist in triaging patients for follow-up care by integrating into primary care systems to provide hip or knee replacement risk scores based on joint pain CTV3 codes. Efforts are also underway to develop explainable methods that visually highlight which parts of the EHR contribute to the model’s risk score. This paper focuses on the development of the TG-CNN model, while the optimization of computational resources and runtime performance required for deployment falls outside the scope of this study. As clinical information systems such as EMIS web are cloud based, computational resources should not be an issue for primary care implementation.

### Clinical utility

Early prediction increases clinical utility for treatment and care planning. However, the 1 year in advance model may also be useful for short term risk, for example in patients experiencing severe pain or reduced mobility, their clinician may wish to observe the likelihood of them needing a replacement sooner.

Integrating this model into clinical workflows can enhance decision-making in primary care by improving referral pathways and optimizing resource use. It could assist general practitioners by identifying patients needing specialist referrals based on longitudinal health data, reducing unnecessary referrals, and acting as an early warning system similar to that of the eFalls tool by detecting frailty earlier via risk thresholding [26]. This may pick up on cases that the general practitioner may not have otherwise noticed. For successful implementation, the model must be embedded in EHRs, be interpretable and continuously monitored for accuracy.

In conclusion, the TG-CNN models developed in this work demonstrate potential for forecasting the likelihood of hip or knee replacement in 1 or 5 years. By identifying individuals at risk, these tools could support proactive clinical interventions aimed at preventing or delaying joint replacement through targeted management strategies. Implementation could enhance operational planning by anticipating future surgical demands and resource allocation. Integrating such predictive models into clinical workflows represents an opportunity to improve patient outcomes and optimize healthcare delivery.

## Supplementary Material

keaf185_Supplementary_Data

## Data Availability

We used anonymized data on individual patients in this study. The ResearchOne data are not distributable under license. A protocol was not prepared for this study, and the study was not registered.
