# Supplementary material for: Primary care prediction of hip and knee replacement 1–5 years in advance using Temporal Graph-based Convolutional Neural Networks (TG-CNNs)
Source: Rheumatology (Oxford). 2025 Apr 3;64(8):4589–98. doi: 10.1093/rheumatology/keaf185 (PMC12316357; doi:10.1093/rheumatology/keaf185)
Supplement: keaf185_Supplementary_Data [file keaf185_supplementary_data.zip › rhe-24-2890-File007.docx]

# Supplementary Materials

## Subgroup Analysis


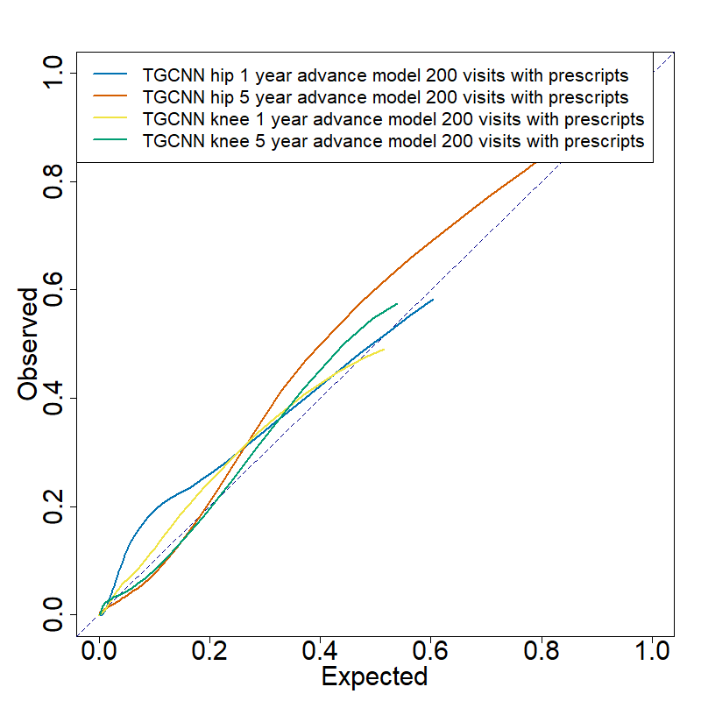

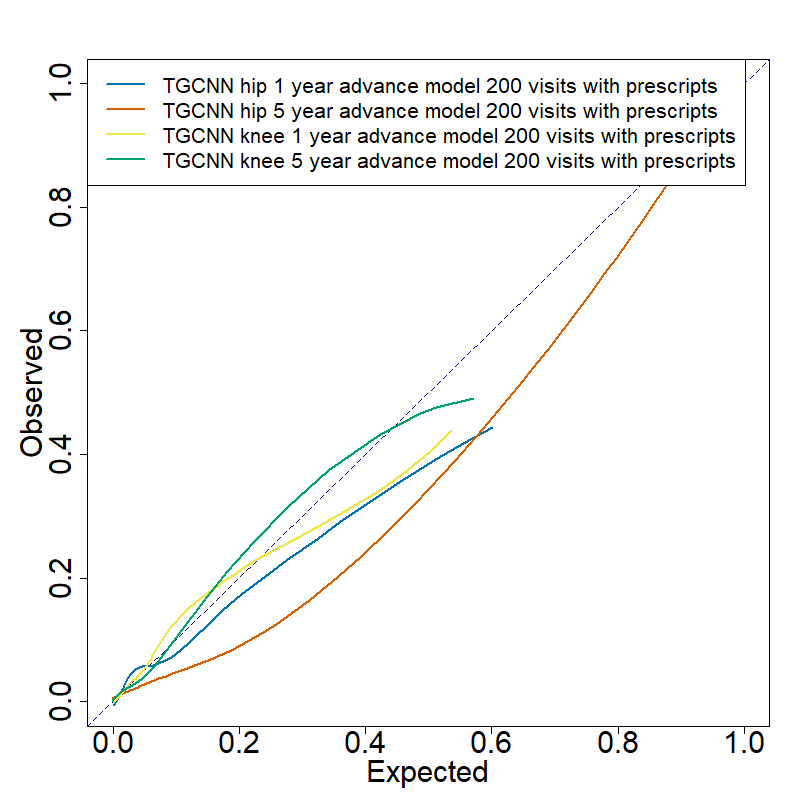


Supplementary Figure S1: Calibration curves for Females (left) and Males (right) in each TG-CNN model.

Females

Males

Supplementary Figure S2: Calibration curves for patients depending on IMD score (where IMD 1 is the most deprived group and IMD 5 is the least deprived quintile) in each TG-CNN model.


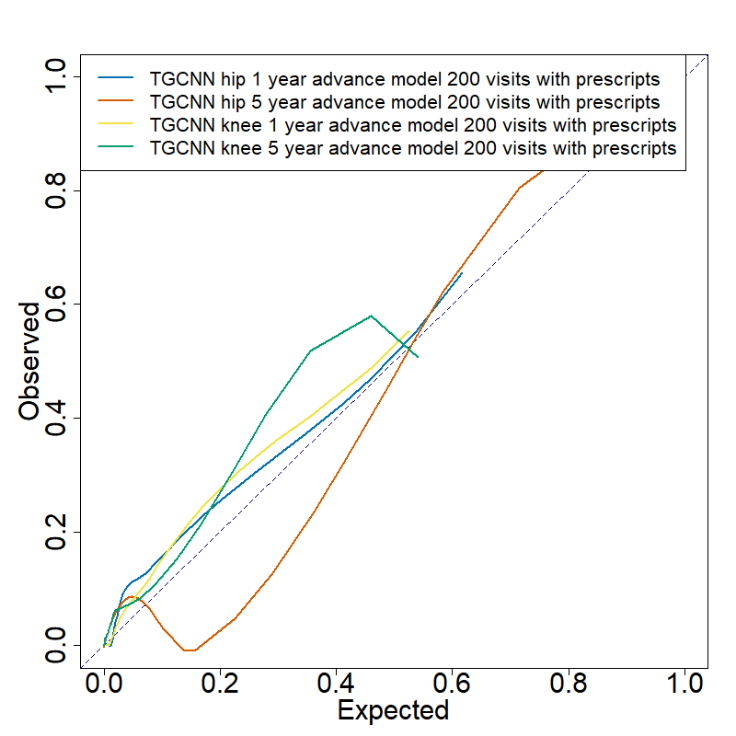

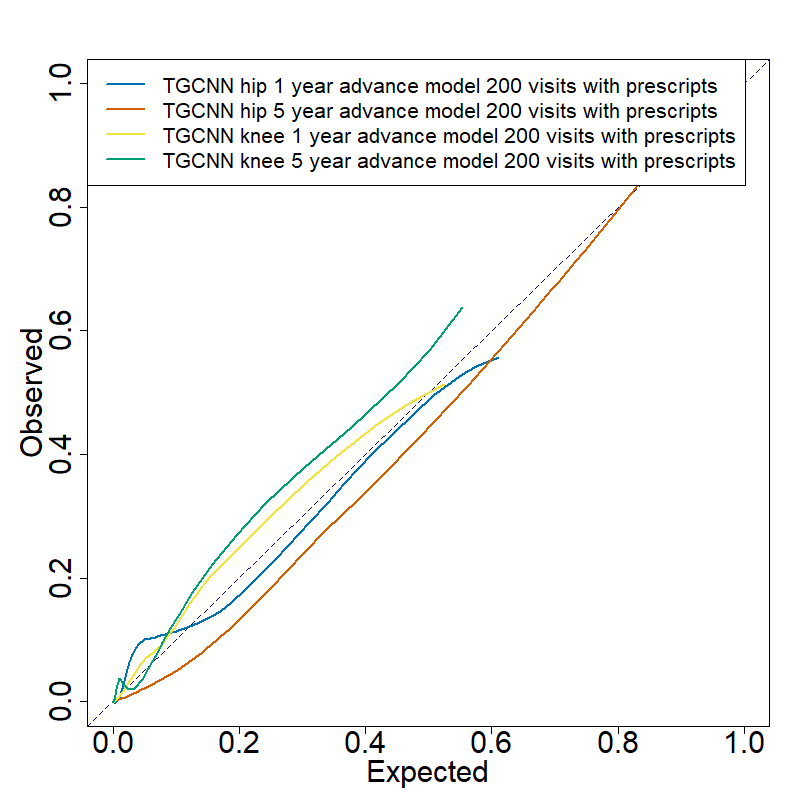

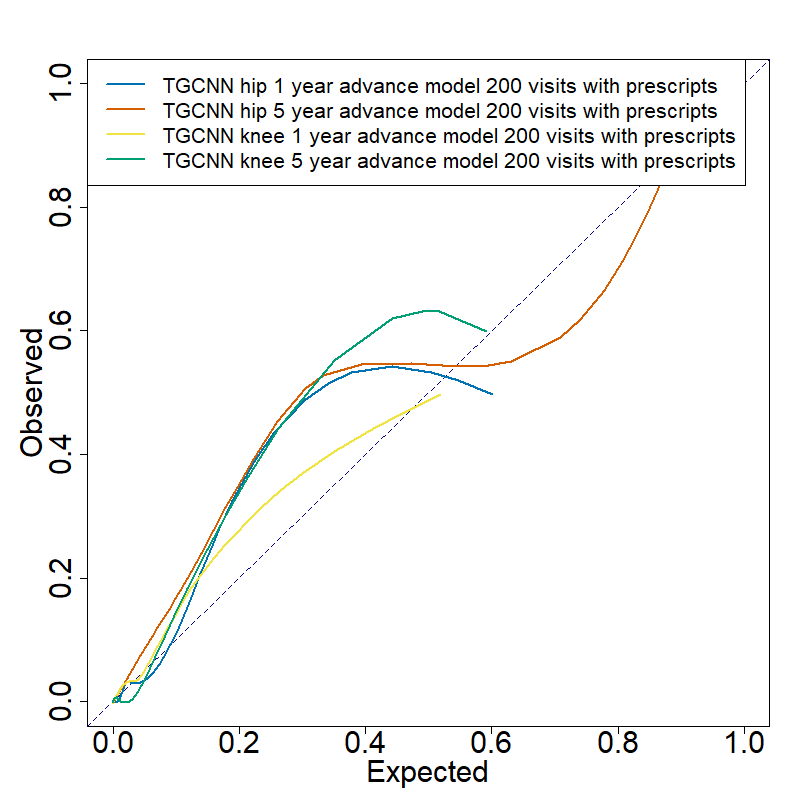

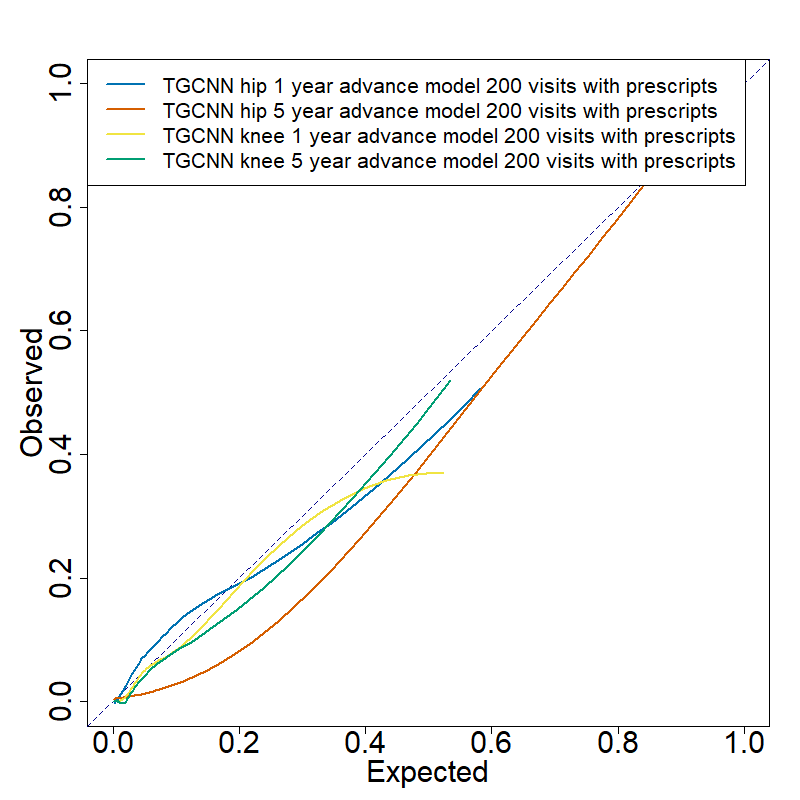

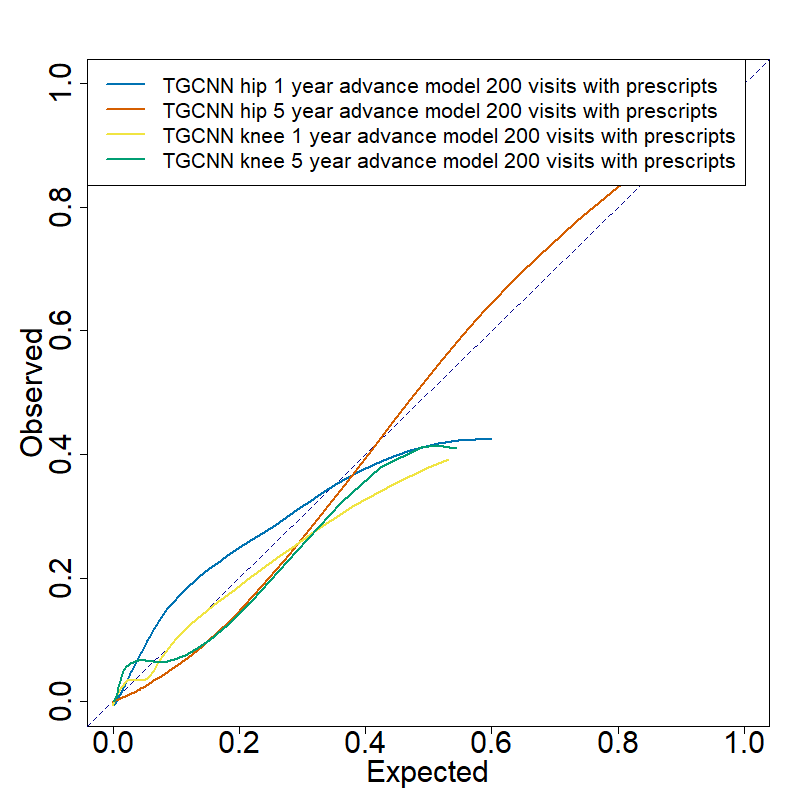


IMD 1

IMD 2

IMD 3

IMD 4

IMD 5

## Extra Dataset Information

Supplementary Table S1: Extra dataset information. Where ‘max # records’ is the maximum number of records a single patient has, and ‘CV’ means cross-validation data set.

|  | **Knee 1 year** | **Knee 5 years** | **Hip 1 year** | **Hip 5 years** |
| --- | --- | --- | --- | --- |
| **# with a revision but no replacement** | 71 | 71 | 161 | 161 |
| **# with a revision before a primary replacement code** | 31 | 31 | 36 | 36 |
| **# patients with mortality** | 1,036 | 1,036 | 1,038 | 1,038 |
| **# patients with no IMD**  **score** | 19 | 19 | 19 | 19 |
| **# patients excluded due to code coverage** | 5 / 150,404 | 5 / 150,404 | 5 / 150,305 | 5 / 150,305 |
| **# replacement patients before windowing** | 10,243 | 10,243 | 12,706 | 12,706 |
| **# replacement patients after windowing** | 9,323 | 6,806 | 11,220 | 7,551 |
| **# patients in CV set (case**  **\| control)** | 7,851 \| 7,851 | 4,949 \| 4,949 | 9,196 \| 9,196 | 5,243 \| 5,243 |
| **# patients in test set (before halving) (case \| control)** | 436 \| 6,959 | 297 \| 6,939 | 1,010 \| 13,673 | 615 \| 13,633 |
| **Full time coverage** | 30,050 / 30,492  (98.55%) | 24,004 / 24,370  (98.50%) | 32,648 / 33,075  (98.71%) | 24,332 / 24,734  (98.38%) |
| **CV set max # records**  **(w/o drugs)** | 210 | 154 | 218 | 154 |
| **CV set mean (SD) #**  **records (w/o drugs)** | 7 (10) | 5 (7) | 6 (9) | 5 (6) |
| **CV set median records**  **(w/o drugs)** | 3 | 2 | 3 | 2 |
| **CV set max # records (w**  **drugs)** | 524 | 303 | 471 | 246 |
| **CV set mean (SD) #**  **records (w drugs)** | 23 (36) | 14 (23) | 17 (31) | 11 (20) |
| **CV set median records**  **(w drugs)** | 5 | 3 | 4 | 3 |
| **Test set max # records**  **(w/o drugs)** | 191 | 399 | 233 | 399 |
| **Test set mean (SD) # records (w/o drugs)** | 18 (15) | 18 (16) | 18 (15) | 18 (16) |
| **Test set median records**  **(w/o drugs)** | 14 | 14 | 13 | 14 |
| **Test set max # records (w drugs)** | 622 | 741 | 889 | 620 |
| **Test set mean (SD) # records (w drugs)** | 47 (54) | 47 (54) | 47 (56) | 49 (55) |
| **Test set median records**  **(w drugs)** | 26 | 27 | 26 | 27 |

## Clinical Code Label Descriptions

Supplementary Table S2: CTV3 Codes (n=46) used for labelling hip replacement.

| **CTV3 Code** | **Description** |
| --- | --- |
| 7K200 | Primary cemented total hip replacement |
| 7K20y | Total prosthetic replacement of hip joint using cement |
| 7K20z | Total prosthetic replacement of hip joint using cement |
| 7K210 | Primary uncemented total hip replacement |
| 7K21y | Total prosthetic replacement of hip joint not using cement |
| 7K21z | Total prosthetic replacement of hip joint not using cement |
| 7K220 | Primary total replacement of hip joint |
| 7K22y | Total replacement of hip |
| 7K22z | Total replacement of hip |
| 7K23. | Thompson hemiarthroplasty of hip joint using cement |
| 7K230 | Primary cemented hemiarthroplasty of hip |
| 7K23y | Arthroplasty of hip joint using cement |
| 7K23z | Arthroplasty of hip joint using cement |
| 7K24. | Prosthetic uncemented hemiarthroplasty of hip |
| 7K240 | Primary uncemented hemiarthroplasty of hip |
| 7K24y | Prosthetic uncemented hemiarthroplasty of hip |
| 7K25. | Partial hip replacement by prosthesis |
| 7K250 | Partial hip replacement by prosthesis |
| 7K25y | Partial hip replacement by prosthesis |
| 7K25z | Partial hip replacement by prosthesis |
| X606J | Total replacement of hip |
| X606K | Partial hip replacement by prosthesis |
| XE08j | Total prosthetic replacement of hip joint using cement |
| XE08k | Primary cemented total hip replacement |
| XE08m | Total prosthetic replacement of hip joint not using cement |
| XE08o | Total replacement of hip |
| XE08r | Thompson hemiarthroplasty of hip joint using cement |
| XE08u | Partial hip replacement by prosthesis |
| XE2n7 | Total replacement of hip |
| XS2Dh | Prosthetic uncemented hemiarthroplasty of hip |
| XaBFE | Prosthetic arthroplasty of the hip |
| XaBFG | Arthroplasty of hip joint using cement |
| XaBFH | Arthroplasty of hip without cement |
| XaBrw | Thompson hemiarthroplasty of hip joint using cement |
| XaF7j | Primary hybrid total replacement of hip joint |
| XaF7k | Primary hybrid total replacement of hip joint |
| XaF7l | Prosthetic hybrid total replacement of hip joint |
| XaMBd | Prosthetic hybrid total replacement of hip joint using cemented acetabular component |
| XaMBe | Primary hybrid total prosthetic replacement of hip joint using cemented acetabular component |
| XaMBj | Prosthetic hybrid total replacement of hip joint using cemented acetabular component |
| XaMBo | Primary hybrid total prosthetic replacement of hip joint using cemented femoral component |
| XaMBu | Prosthetic hybrid total replacement of hip joint using cemented femoral component |
| XaMC4 | Prosthetic hybrid total replacement of hip joint using cement |
| XaMC5 | Prosthetic hybrid total replacement of hip joint using cement |
| XaMCB | Prosthetic hybrid total replacement of hip joint using cement |
| 7K21. | Total prosthetic replacememt hip joint not using cement |

Supplementary Table S3: CTV3 Codes (n=33) used for labelling knee replacement.

| **CTV3 Code** | **Description** |
| --- | --- |
| 7K30. | Cemented knee arthroplasty (\& total (\& named variants)) |
| 7K300 | Primary cemented total knee replacement |
| 7K30y | Total prosthetic replacement of knee joint using cement OS |
| 7K30z | Total prosthetic replacement of knee joint using cement NOS |
| 7K31. | Arthroplasty knee joint without cement (\& total) |
| 7K310 | Primary uncemented total knee replacement |
| 7K31y | Total prosthetic replacement knee joint not using cement OS |
| 7K31z | Total prosthetic replacement knee joint not using cement NOS |
| 7K32. | Other arthroplasty knee joint (\& total) |
| 7K320 | Primary total knee replacement NEC (\& hybrid) |
| 7K32y | Other total prosthetic replacement of knee joint OS |
| 7K32z | Other total prosthetic replacement of knee joint NOS |
| 7K37. | Cemented unicompartmental knee replacement |
| 7K370 | Primary cemented unicompartmental knee replacement |
| 7K38. | Uncemented unicompartmental knee replacement |
| 7K380 | Primary uncemented unicompartmental knee replacement |
| 7K39. | Hybrid unicompartmental knee replacement |
| 7K390 | Primary hybrid unicompartmental knee replacement |
| X606N | Arthroplasty of the knee |
| X606O | Prosthetic total arthroplasty of the knee |
| X606P | Prosthetic unicompartmental arthroplasty of the knee |
| X606Q | Prosthetic medial unicompartmental arthroplasty of the knee |
| XE07f | Knee arthroplasty (\& replacement) |
| XE08w | Total prosthetic replacement of knee joint using cement |
| XE08y | Total prosthetic replacement of knee joint not using cement |
| XE090 | Other total prosthetic replacement of knee joint |
| XE091 | Primary hybrid total knee replacement NEC |
| XaBFJ | Prosthetic arthroplasty of knee |
| XaBFK | Arthroplasty of knee using cement |
| XaBFM | Arthroplasty of knee without cement |
| XaOPm | Unicompartmental knee replacement NOS |
| XaPtO | Hybrid prosthetic replacement of knee joint using cement |
| XaPtP | Primary hybrid prosthetic replacement knee joint using cement |

## T-testing

Supplementary Table S4: T-statistics, degrees of freedom and p-values when comparing TG-CNN models with and without prescriptions and comparing the TG-CNN models statistical difference to Logistic regression AUROC and AUPRC.

| **Comparison** | **t-stat using AUROC** | **t-stat using AUPRC** | **Degrees of Freedom** | **p-value** |
| --- | --- | --- | --- | --- |
| **Hip 1 year TG-CNN without vs with prescriptions** | 1361.186 | 720.583 | 14680 | 0.0 |
| **Hip 5 years TG-CNN without vs with prescriptions** | 401.095 | 261.647 | 14246 | 0.0 |
| **Knee 1 year TG-CNN without vs with prescriptions** | 953.907 | 447.052 | 7392 | 0.0 |
| **Knee 5 years TG-CNN without vs with prescriptions** | 493.155 | 333.347 | 7232 | 0.0 |
| **Hip 1 TG-CNN with prescriptions vs logistic regression** | -292.930 | -175.841 | 14680 | 0.0 |
| **Hip 5 TG-CNN with prescriptions vs logistic regression** | -104.022 | 594.008 | 14246 | 0.0 |
| **Knee 1 TG-CNN with prescriptions vs logistic regression** | -133.044 | -36.447 | 7392 | 0.0 |
| **Knee 5 TG-CNN with prescriptions vs logistic regression** | -123.646 | -78.364 | 7232 | 0.0 |

## Literature Review Method

When reviewing the current literature in the area the following research question was asked: What predictive methods and models are used to assess an individual's future risk of primary hip or knee replacement before secondary care referral?

Ovid MEDLINE®, Scopus, Web of Science and IEEE Xplore were searched on 25/10/2024 for articles predicting hip and knee replacement risk using the following search string:

("individual risk" OR "personali$ed risk" OR "future risk” OR “replace* risk”) AND (“machine learning” OR "statistical models" OR algorithms OR "predict*" OR "model" OR "risk model" OR "risk prediction" OR “AI” or “artificial intelligen*”) AND (“THR” OR “THA” OR “hip arthroplast*” OR “TKR” OR “TKA” OR “knee arthroplast*” OR “hip replace*” OR “knee replace*”)

Titles and abstracts were screened first, then full-texts were screened looking for papers that met the research question criteria. Papers were included if they were written in English and gave a future risk score for hip or knee replacement. Papers were excluded if risk prediction was for other outcomes rather than primary hip replacement, e.g., revision, mortality. Papers were also excluded if they explored associations or thresholding rather than developing or validating predictive models at an individual level.

Grey literature and literature reviews were included in the search to get a wide overview of current methodologies being explored. Relevant papers were extracted from reviews, using the snowballing technique.

## Literature Review Results

Inputting the search string into databases resulted in 1,613 papers being returned (Scopus (N=612), Ovid (N=369), Web of Science (N=608), IEEE Xplore (N=24)). 783 papers were removed during deduplication, leaving 830 papers for title and abstract screening. After title screening 91 papers were left. After abstract screening 6 papers remained. 1 of these papers were literature reviews (32), from which snowballing of references was performed but no extra research question relevant papers were found, totalling 5 full papers to screen. See Figure S3 for the PRISMA flowchart (33). In total 5 papers discussed methods to give personalised future hip or knee replacement risk.

Many of the non-eligible papers were predicting risks of complications, revisions, readmissions, mortality, implant size, and other outcomes following TJR, rather than predicting TJR risk.


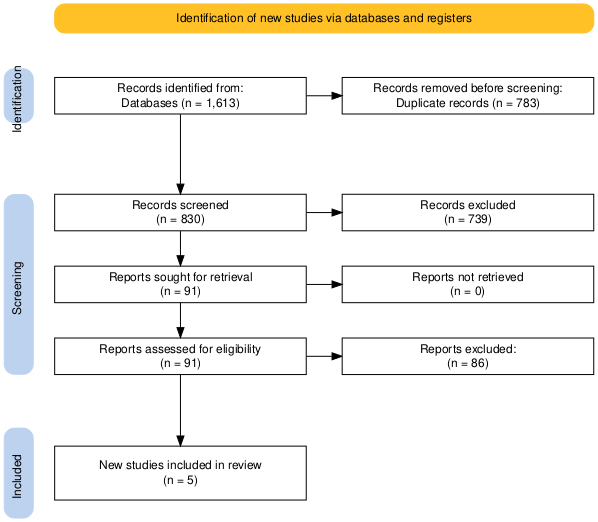


Supplementary Figure S3: PRISMA flowchart for systematic search of papers predicting hip and knee replacement risk using primary care data.

## Model Architecture, Development and Validation Processes


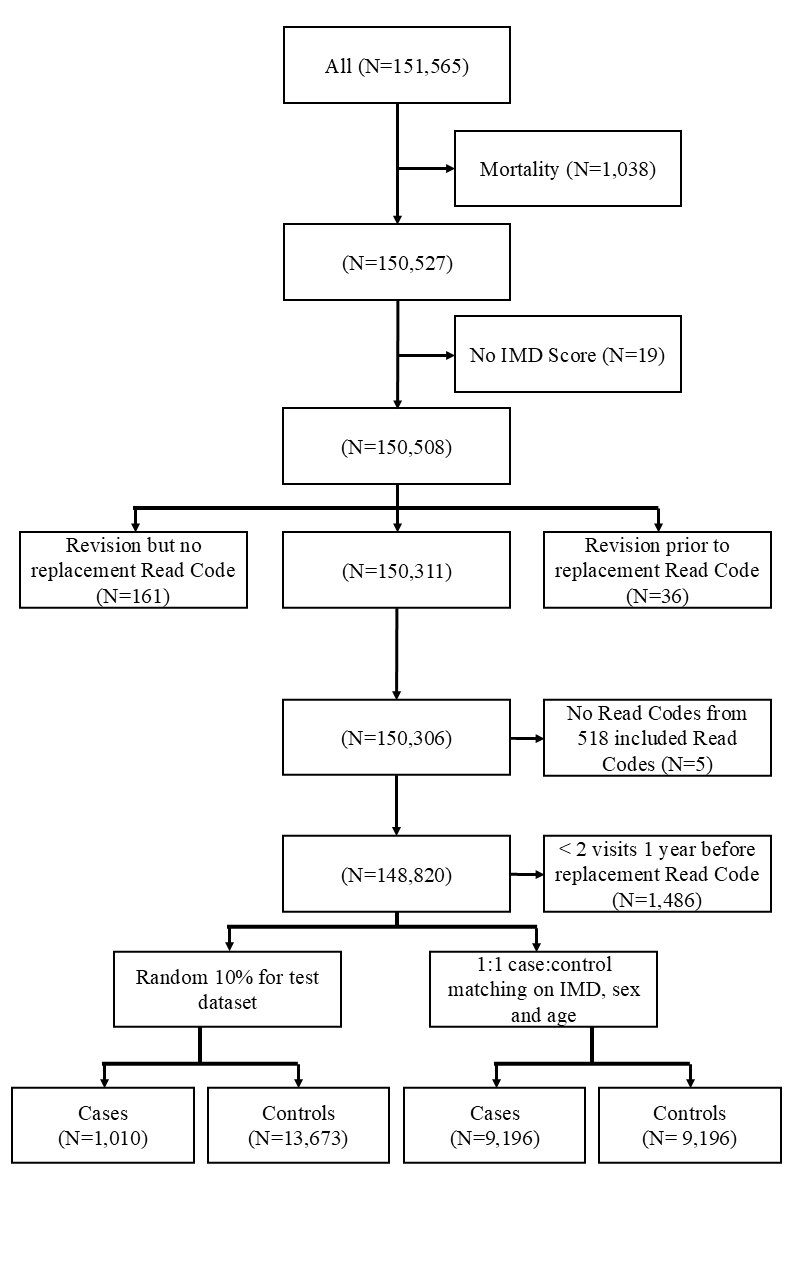


Supplementary Figure S4: Patient inclusion criteria for hip 1 year in advance cohort. Following cohort generation the model is trained using 5-fold cross validation on the 1:1 case:control matched patient. The model is then recalibrated on half of the test dataset, and validated on the unseen half of the test dataset using the recalibrated model.


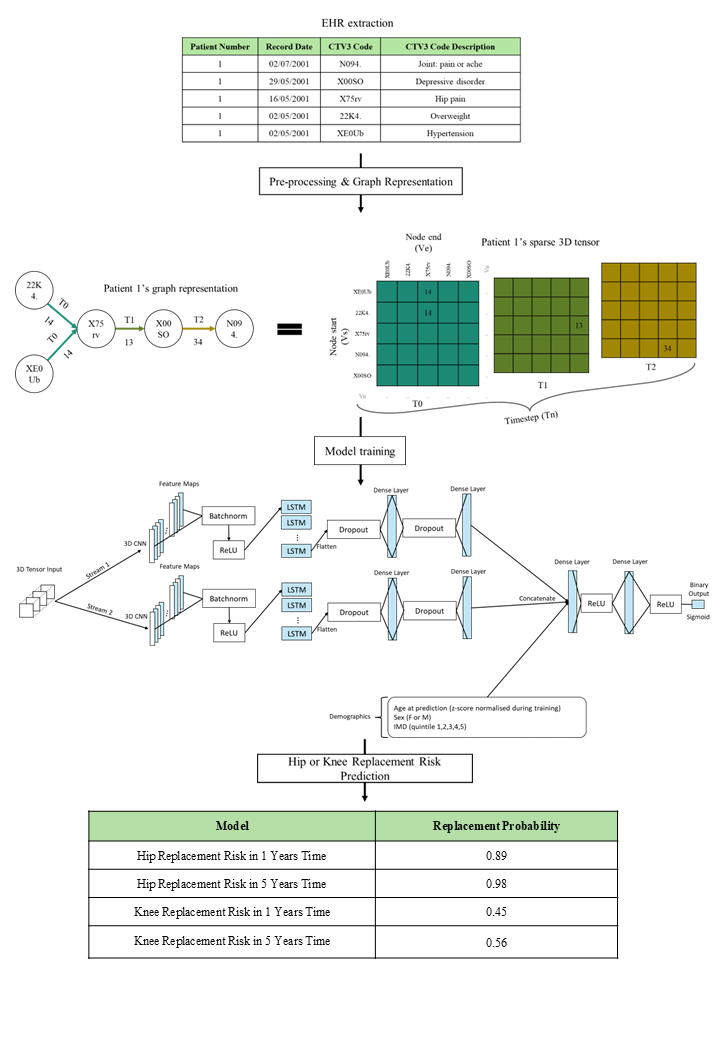


Supplementary Figure S5: Data to prediction process of TG-CNN model with model architecture summary. After each patient's EHR is converted to a 3-tensor (graph representation) a neural network architecture is created which applies convolutions over the time axis of the tensor. The temporal graph representation, sized at $512\times512\times100$, undergoes processing through the 3D CNN layer to extract Clinical Code sequences and elapsed time patterns. The CNN output is then flattened, followed by batch normalisation for faster convergence, and subsequent layers include a Leaky rectified linear unit (ReLU), LSTM, dropout, dense layers, and concatenation with demographic features. The model employs binary cross-entropy loss with sigmoid for the binary target.


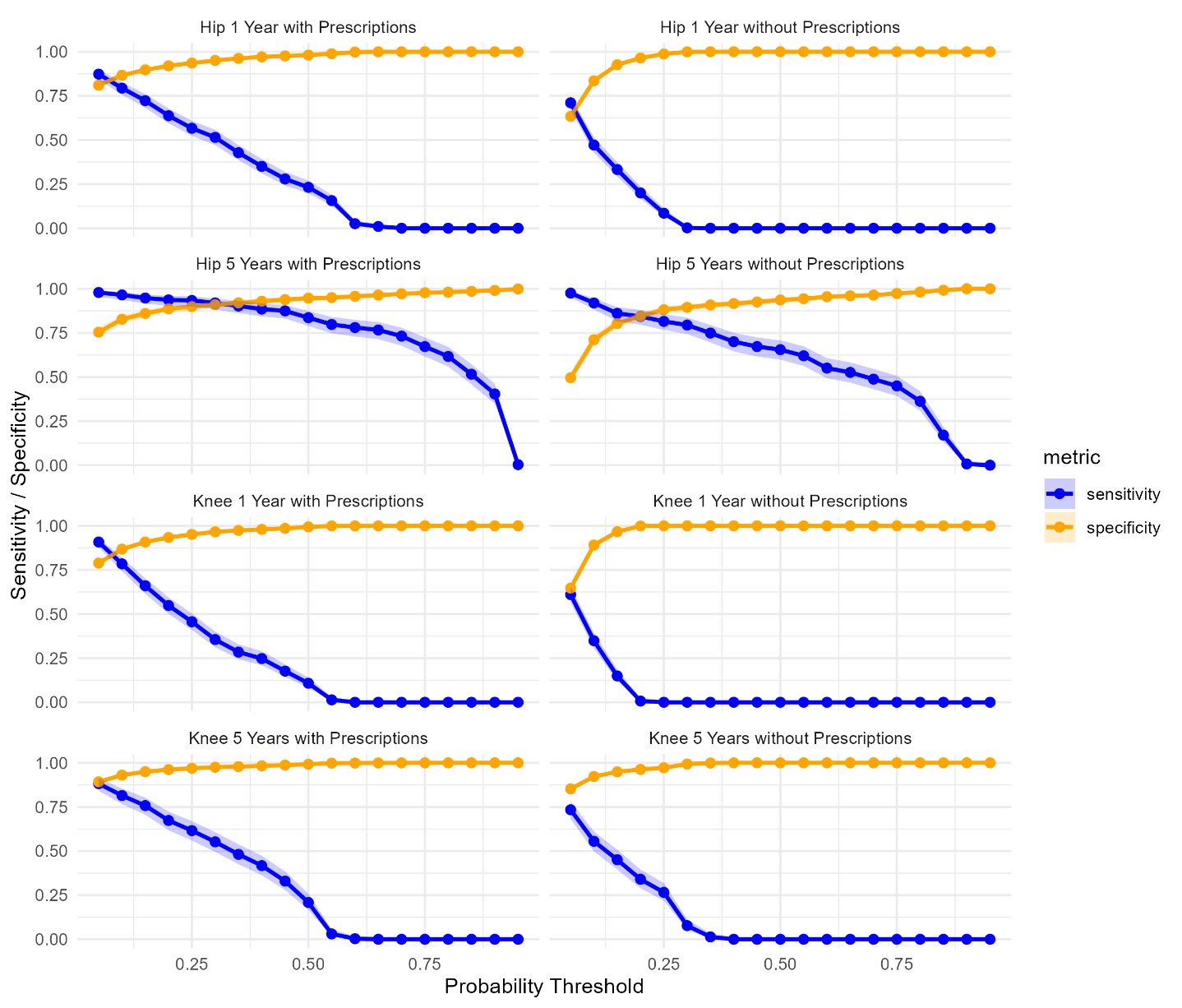


Supplementary Figure S6: Sensitivity and specificity plots for the TG-CNN models at each probability threshold. The hip 5 years in advance models have the best sensitivity and specificity across the thresholds.


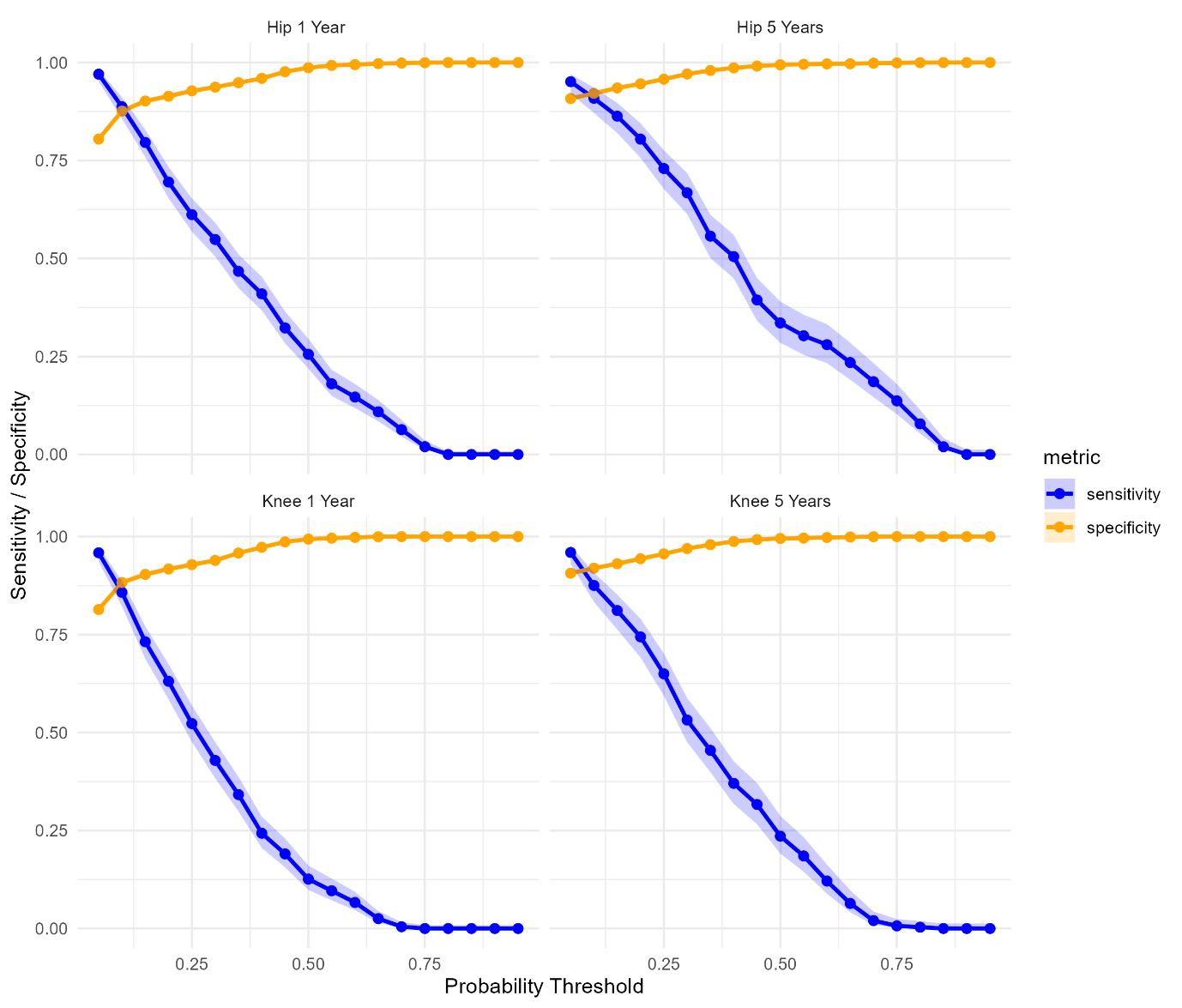


Supplementary Figure S7: Sensitivity and specificity plots for the Logistic regression models at each probability threshold.
